# Characteristics of patients with neonatal intrahepatic cholestasis caused by citrin deficiency in China: long-term follow-up outcomes

**DOI:** 10.1016/j.jped.2026.101553

**Published:** 2026-05-19

**Authors:** Lingli Chen, Jingan Lou, Youyou Luo, Youhong Fang, Mingfang Sun, Jindan Yu

**Affiliations:** Department of Gastroenterology, Children’s Hospital, Zhejiang University School of Medicine, National Clinical Research Center for Children and Adolescents’ Health and Diseases, Hangzhou, China

**Keywords:** Citrin deficiency, *SLC25A13* gene, Neonatal intrahepatic cholestasis caused by citrin deficiency, Failure to thrive and dyslipidemia caused by citrin deficiency

## Abstract

**Objective:**

Citrin deficiency (CD) is an autosomal recessive disease caused by mutations in the *SLC25A13* gene. This study aimed to expand the current body of data on Chinese patients with neonatal intrahepatic cholestasis caused by citrin deficiency (NICCD) by analyzing their clinical characteristics, genetic mutation spectrum, and long-term follow-up outcomes.

**Methods:**

From May 2013 to April 2025, 60 children diagnosed with NICCD were enrolled in this retrospective study**.** Related data were obtained from medical records.

**Results:**

Among 60 patients, common presentations included elevated aspartate aminotransferase (100%), infantile cholestasis (95.0%), elevated citrulline (96.7%), hyperlactatemia (93.3%), hypoproteinemia (81.7%), coagulation dysfunction (60.0%), hyperammonemia (48.3%) and chubby face (36.7%). Twenty-eight *SLC25A13* variants were detected, with c.852_855delTATG (42.7%), IVS16ins3kb (15.4%) and c.615+5G>A (10.3%) being the most frequent. All patients were fed lactose-free milk powder enriched with medium-chain triglycerides (MCT) after diagnosis or suspected diagnosis. Ten patients were lost to follow-up. Among 50 followed patients, 30 were followed for > 5 years. Twenty-four patients showed typical dietary features. After discharge, 11 had hypoglycemic episodes, 5 had growth retardation, 11 had dyslipidemia and 3 progressed to failure to thrive and dyslipidemia caused by citrin deficiency (FTTDCD). All patients remained in stable condition.

**Conclusion:**

Patients with neonatal intrahepatic cholestasis caused by citrin deficiency present with a variety of clinical manifestations. c.852_855delTATG, IVS16ins3kb and c.615+5G>A are the mutation hotspots of the *SLC25A13* gene in Zhejiang, China. Early intervention leads to a good prognosis.

## Introduction

Neonatal cholestasis is a prevalent disorder in pediatrics, primarily caused by biliary obstruction, genetic metabolic disorders, and infections [1,2]. Its complex etiology poses significant challenges for early diagnosis and may lead to adverse outcomes. Citrin deficiency (CD) is a common cause of infantile cholestasis among inborn errors of metabolism. *SLC25A13* is the pathogenic gene of CD, which encodes the citrin protein. Citrin, also known as aspartate/glutamate carrier 2 (AGC2), is a mitochondrial inner membrane protein predominantly expressed in the liver that plays a vital role in energy metabolism. Additionally, it is a crucial component of the malate-aspartate nicotinamide adenine dinucleotide (NADH) shuttle system. Its deficiency disrupts the urea cycle, hepatic glycolysis, gluconeogenesis, and de novo lipogenesis, leading to a series of metabolic disturbances [3, 4, 5]. CD presents with three age-dependent clinical phenotypes: neonatal intrahepatic cholestasis caused by citrin deficiency (NICCD), post-NICCD (including failure to thrive and dyslipidemia caused by citrin deficiency, FTTDCD), and adolescent and adult citrin deficiency (AACD) [6, 7, 8, 9].

NICCD mainly presents a series of clinical symptoms, such as cholestasis and elevated liver enzymes, and is often accompanied by various metabolic abnormalities, including citrullinemia, hypoglycemia, galactosemia and hyperammonemia [7]. The primary treatment involves early initiation of a lactose-free, medium-chain triglyceride (MCT)-enriched formula, combined with supportive hepatoprotective therapy. Most patients achieve significant symptomatic improvement within their first year of life. However, NICCD carries a risk of adverse long-term outcomes. These include post-infancy growth retardation, progression to cirrhosis requiring transplantation, and mortality due to severe complications [10].

As there are no universally accepted diagnostic criteria for NICCD, genetic testing is essential for confirmation. The accurate application of molecular diagnostic techniques is therefore critical for enabling timely diagnosis and effective intervention [11]. The *SLC25A13* gene, located at chromosome 7q21.3 and responsible for citrin deficiency, consists of 18 exons and 17 introns, encoding a 675-amino acid protein [7]. More than 140 pathogenetic/likely pathogenetic variants have been reported [6]. Population-based studies indicate that the carrier frequency of pathogenic *SLC25A13* variants in China is 1/45, and the predicted disease prevalence is approximately 88,000 affected individuals [12,13]. However, only a few hundred patients have been reported so far, which means the majority of patients have been either undiagnosed or misdiagnosed. Insufficient awareness of the disease remains the primary barrier to diagnosis. Therefore, this study aims to clarify the clinical features and genetic characteristics of NICCD, as well as prognosis, to enrich the present data of citrin deficiency in China and provide support for further improving the diagnosis and treatment of this disease.

## Methods

### Subjects

This is a retrospective analysis. From May 2013 to April 2025, 60 children diagnosed with neonatal intrahepatic cholestasis caused by citrin deficiency in the department of gastroenterology, Children’s Hospital, Zhejiang University School of Medicine, were selected as the study subjects. Inclusion criteria were as follows: (1) age of onset < 1 year; (2) diagnosed with intrahepatic cholestasis and/or elevated citrulline; (3) carrying a homozygous or heterozygous variant of the *SLC25A13* gene.

The study was carried out in line with the Declaration of Helsinki and was approved by the Medical Ethics Committee of the Children’s Hospital, Zhejiang University School of Medicine (No.2025-IRB-0536).

### Clinical data collection

General information(including gender, age at consultation, birth history, onset date and consultation date, family history, childbirth history, height and weight follow-ups as well as height-for-age z-score (HAZ), weight-for-age z-score (WAZ) (<5y), and body mass index-for-age z-score (BAZ) (5–18y) calculated by the WHO Anthro software at diagnosis and outpatient follow-up), clinical manifestations, physical signs, laboratory examinations, imaging examinations, genetic results, treatment and follow-up information of the patients were collected from medical records. Patients with citrin deficiency were regularly monitored for clinical manifestations, liver function, blood ammonia, plasma amino acids, blood lipid profile, blood glucose, growth status, dietary compliance, and treatment response. Abnormal parameters were assessed monthly; once normalized, they were evaluated every 3 months during the first year of life and every 6–12 months thereafter.

### Metabolic index detection

Dried blood spot (DBS) samples were collected from patients via heel stick or finger stick puncture. Amino acid and acylcarnitine profiles were determined by MS/MS using the NeoBase Non-derivatized MS/MS Kit (PerkinElmer, Finland). Briefly, 100 μL of working solution containing internal standards was added to the U-bottom plates. After vibrating at 700 rpm and incubation at 45 °C for 45 min, 75 μL liquor was transferred to V-bottom plates. Following standing at room temperature for 2 h, 25 μL of liquor was injected into the tandem mass spectrometer for metabolic analysis. Low and high internal quality controls were used for quality control.

### Molecular testing

Genomic DNA was extracted from peripheral blood samples of 60 NICCD patients and their family members. Subsequent to gene capture sequencing, raw data processing and comprehensive bioinformatics analyses were conducted. For single-nucleotide variants (SNVs), pathogenicity and evolutionary conservation predictions were conducted using SIFT, PolyPhen-2 and MutationTaster bioinformatics tools. The candidate variant loci screened through the analysis were verified by PCR and Sanger sequencing, and co-segregation verification was performed among family members. Among these patients, 24 patients were subjected to whole-exome sequencing (WES), 13 patients via multigenic panels related to cholestasis and 23 patients via *SLC25A13* gene detection.

### Statistical analysis

Statistical analyses were conducted using the Statistical Package for the Social Sciences version (SPSS) 22.0 (IBM Corp., Armonk, NY, USA). Categorical variables are presented as counts and percentages. For continuous variables, those following a normal distribution are summarized as mean ± standard deviation (SD), while nonnormally distributed continuous variables are expressed as median (minimum ∼ maximum).

## Results

### Clinical manifestations of the patients

Four patients (6.7 %) had a positive family history, with one elder brother, one younger brother, and two elder sisters carrying the same gene mutations. Abdominal ultrasound detected hepatic echogenicity enhancement in 23 patients. Among 60 patients, 57 patients (95.0 %) were diagnosed with infantile cholestasis, 49 patients (81.7 %) had hypoproteinemia, 21 patients (35.0 %) had elevated alanine aminotransferase (ALT), 60 patients (100.0 %) had elevated aspartate aminotransferase (AST), 90.0 % of patients had AST/ALT > 2. Hyperlactatemia was present in 56 patients (94.9 %), with 19 patients having severe hyperlactatemia. Thirty-six patients (60 %) had coagulation dysfunction of varying degrees, among whom 8 patients had prothrombin time exceeding 20 s. Twenty-nine patients (48.3 %) had hyperammonemia. Twelve patients (20.0 %) experienced hypoglycemic episodes during hospitalization. Details were provided in Table 1, Table 2.

**Table 1:** Demographic, baseline, and clinical symptoms of 60 NICCD patients at diagnosis.

	Number of patients	Percentage
Demographic and baseline characteristics of the patients		
**Gender**		
Male	31	51.7 %
Female	29	48.3 %
**Birth history**		
Term infants	57	95.0 %
Preterm infants	3	5.0 %
**Birth weight**		
< 2500 *g* 2500–2999 *g*	10 27	16.7 % 45.0 %
3000–4000 *g* > 4000 *g*	22 1	36.7 % 1.7 %
**Age of onset**		
< 1 month	31	51.7 %
1–3months	24	40.0 %
> 3 months	5	8.3 %
**Age of diagnosis**		
< 3 months	43	71.7 %
3–6 months	16	26.7 %
> 6 months	1	1.7 %
**Geographic distribution**		
Zhejiang province	50	83.3 %
Other cities in China	10	16.7 %
**HAZ**		
<−2SD	21	35.0 %
−2SD∼−1SD	22	36.7 %
−1SD∼1SD	15	25.0 %
1SD∼2SD	2	3.3 %
>2SD	0	0 %
**WAZ**		
<−2SD	16	26.7 %
−2SD∼−1SD	27	45.0 %
−1SD∼1SD	16	26.7 %
1SD∼2SD	1	1.7 %
>2SD	0	0 %
Clinical symptoms at diagnosis		
Jaundice	60	100.0 %
Chubby face	22	36.7 %
Pale stools	16	26.7 %
Hepatomegaly	6	10.0 %
Splenomegaly	3	5.0 %
Lenticular opacity	1	1.7 %

**Table 2:** Laboratory test results of 60 NICCD patients before dietary therapy.

Laboratory test indexes	Median (Minimum ∼ Maximum)	Reference range
Albumin(g/L)	30.7(23.6∼43.1)	32.0∼52.0
Total bilirubin(μmol/L)	142.3(34.7∼282.1)	5.0∼21.0
Conjugated bilirubin(μmol/L)	62.1(13.5∼156.4)	0∼5.1
Unconjugated bilirubin(μmol/L)	74.4(19.1∼160.0)	1.0∼20.0
Alanine transaminase(U/L)	43.0(20.0∼156.0)	<50.0
Aspartate transaminase(U/L)	137.0(70.0∼359.0)	15.0∼60.0
γ-Glutamyl transferase(U/L)	200.0(85.0∼609.0)	5.0∼19.0
Alkaline phosphatase(U/L)	1023.0(43.0∼2915.0)	42.0∼362.0
Serum bile acids(μmol/L)	239.4(112.9∼648.8)	0∼12.0
Total cholesterol(mmol/L)	5.0(1.8∼9.7)	3.0∼5.7
Triacylglycerol(mmol/L)	1.6(0.4∼5.8)	<1.7
AFP (ng/ml)		
< 3 months	222,559.0(12,365.0∼>484,000)	No range
3 to < 6 months	88,733.0(21,035.0∼300,942.0)	5.15∼274.7
6 to < 12 months	One patient not done	2.7∼148.2
≥ 12 months	No patient	0∼20.0
Ammonia(μmol/L) (umol/L)		
	80.0(22.0∼171.0)	9.0∼30.0(before 2024.7)
	77.0(62.0∼122.0)	18.0∼72.0(2024.7-present)
Lactate(mmol/L)	4.2(1.5∼9.3)	0.5∼1.6
Prothrombin time(s)	15.7(10.6∼25.7)	9.0∼14.0
Activate partial thrombin activity(s) time(s)	51.6(27.6∼93.8)	23.0∼38.0
International normalized ratio	1.4(0.9∼2.1)	0.8∼1.2

### Blood tandem mass spectrometry detection

Of the 58 patients who underwent MS/MS newborn screening (NBS), only 3 (5.2 %) were found to have elevated citrulline levels. Amino acid profiles revealed elevated citrulline (96.7 %), methionine (83.6 %), arginine (78.8 %), and tyrosine (11.8 %). Details were provided in Table 3. Acylcarnitine profiles revealed increased concentrations of free carnitine (71.2 %) and long-chain acylcarnitines (78.4 %).

**Table 3:** Amino acids of 60 NICCD patients at diagnosis.

Amino acid	Median (Minimum ∼ Maximum)	Reference range
Citrulline (μmol/L)	304.12(26.79∼888.66)	7.13∼37
Methionine (μmol/L)	134.1(16.09∼784.14)	7.18∼41.35
Arginine (μmol/L)	85.71(12.08∼200.01)	2.54∼50
Tyrosine (μmol/L)	152.17(16.49∼355.34)	34.5∼280
Ornithine (μmol/L)	246.75(102.94∼403.31)	52.09∼386.6
Glycine (μmol/L)	252.26(73.77∼423.65)	246.57∼1283
Phenylalanine (μmol/L)	50.42(15.68∼84.03)	23.3∼100
Alanine (μmol/L)	220.91(60.34∼419.97)	136.5∼650
Valine (μmol/L)	124.82(59.3∼257.52)	52.7∼305
Leucine (μmol/L)	137.85(53.2∼260.48)	75.7∼316
Proline (μmol/L)	197.94(16.13∼425.24)	97.2∼440.3

### Gene variation

**Figure 1:**
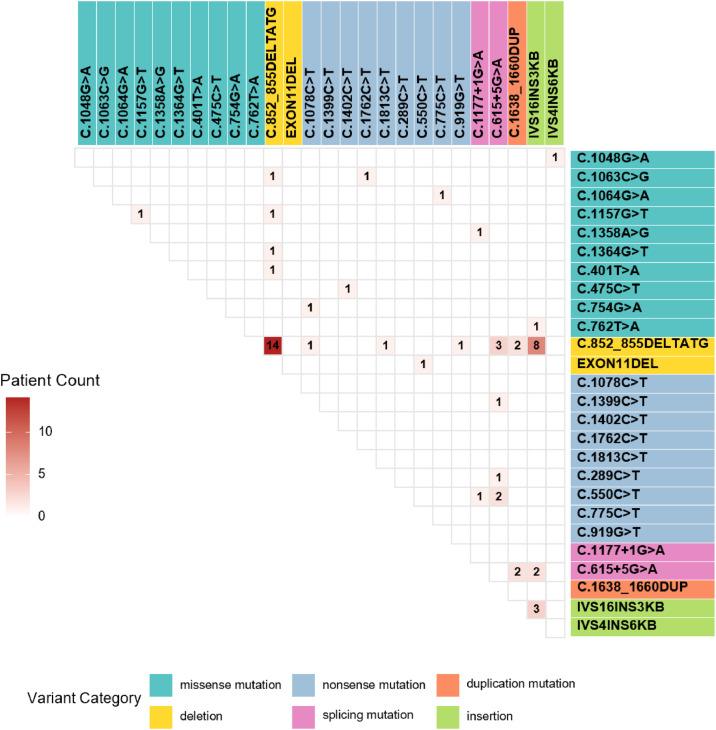
Genetic landscape of *SLC25A13* variants in 55 NICCD patients.

Compound heterozygous mutations were detected in 38 patients, with the mutations inherited from their parents. Among them, one patient harbored three mutation variants: c.1067T>A and c.1046T>*C* were paternally inherited, whereas c.1157G>T was maternally inherited. Eighteen patients carried homozygous mutations, with one mutant allele inherited from each parent. Four patients carried single heterozygous mutations. Among them, two harbored c.852_855delTATG of paternal origin, one harbored c.615+5G>A of maternal origin, and one harbored IVS16ins3kb, which was a de novo mutation. Fifty-five patients with two gene variants were shown in Figure 1. Genetic analysis of 60 NICCD patients identified 28 types of *SLC25A13* mutations, c.852_855delTATG (42.7 %) being most prevalent, followed by IVS16ins3kb (15.4 %), c.615+5G>A (10.3 %), c.1638_1660dup (3.4 %) and c.550C>T (3.4 %). The spectrum comprised two deletions, two insertions, 12 missense mutations, two splicing mutations, nine nonsense mutations and one duplication.

### Treatment

In the treatment of NICCD patients, interventions were implemented across the following several aspects. Symptomatic and supportive treatment was given before diagnosis and discontinued after confirmation. Ursodeoxycholic acid (dosage: 10mg/kg, qd or q12h) and/or ademetionine 1,4-butanedisulfonate were used to improve the cholestatic features in all patients. Glycyrrhizin and/or glucuronolactone were used to improve abnormal liver function in 27 patients. Albumin was administered to 23 patients with serum albumin levels < 30 g/L. Potassium magnesium aspartate was used for reducing blood ammonia in 10 patients. Supplementation of coagulation factors was used for the treatment of coagulation disorders in 28 patients. All patients received lactose-free and medium-chain triglyceride-enriched formula after diagnosis or suspected diagnosis. After complementary feeding was introduced, a dietitian developed a personalized low-carbohydrate, high-fat, and high-protein diet for each patient during follow-up visits. Fat-soluble vitamins were supplemented in all patients.

### Long-term follow-up outcomes

Of the 60 NICCD patients, 10 patients were lost to follow-up. Among the remaining 50 patients, 30 had a follow-up period exceeding 5 years, with the longest follow-up duration reaching 12 years. Forty-seven patients had their bilirubin levels return to normal within six months after discharge from the hospital, of whom 31 recovered within three months after discharge. Another two patients achieved normal bilirubin levels between six months and one year after discharge. One patient did not return for a follow-up examination for a long time after discharge, so the time of recovery to normal remained unknown. Twenty-four patients (48.0 %) preferred protein- and lipid-rich foods and avoided carbohydrate-rich foods. Five patients presented with growth retardation, including two with HAZ < −2 and three with BAZ < −2. Eleven patients still experienced hypoglycemic episodes after discharge. Eleven patients presented with dyslipidemia. Three patients developed FTTDCD. One of them underwent a growth hormone stimulation test, which indicated partial growth hormone deficiency, and had received growth hormone replacement therapy for approximately 3 years. One patient developed fatty liver. One patient developed severe vomiting and pallor after oral administration of a high dose of carbohydrate-rich foods. During the follow-up period, no patients developed severe complications. Details were shown in Table 4.

**Table 4:** Long-term outcomes in 50 patients with NICCD at the last follow-up.

	Follow-up < 5 years(n)	Follow-up:5–10 years(n)	Follow-up > 10 years(n)
**Gender**			
Male	12	7	6
Female	8	9	8
**HAZ**			
<−2SD	1	1	0
−2SD∼−1SD	7	3	3
−1SD∼1SD	12	11	9
1SD∼2SD	0	1	1
> 2SD	0	0	1
**WAZ(<5y)/ BAZ(5–18y)**			
< −2SD	0	1	2
−2SD∼−1SD	0	6	4
−1SD∼1SD	16	6	7
1SD∼2SD	4	2	1
> 2SD	0	1	0
**Dietary habits**			
Typical dietary characteristics	8	10	6
Preferred high-protein, high-fat foods only	1	2	6
Disliked carbohydrates only	1	0	0
No special dietary habits	10	4	2
**Hypoglycemic episodes after discharge**	6	3	2
**Dyslipidemia**	5	4	2
**FTTDCD**	0	2	1
**Total patients**	20	16	14

SD, standard deviation; n, numbers; HAZ, height for age z-score; WAZ, weight for age z-score; BAZ, body mass index for age z-score.

## Discussion

The present study delineates the clinical and genetic characteristics of 60 NICCD patients, among whom 50 were followed up, with the longest follow-up duration reaching 12 years. NICCD patients present with a wide range of clinical manifestations. Three major hotspot mutations in *SLC25A13* were identified in NICCD patients from Zhejiang, China: c.852_855delTATG, IVS16ins3kb, and c.615+5G>A. Following treatment with lactose-free and MCT-enriched formula, patients achieved rapid clinical resolution. Three patients progressed to FTTDCD. All patients remained clinically stable during follow‑up, suggesting that early intervention contributes to a favorable prognosis.

It is reported that chubby face features, along with AST/ALT >2, direct bilirubin(DB)/total bilirubin(TB)<0.67, and a standard deviation score for α-fetoprotein of 4 or greater, might serve as useful clinical indicators for diagnosing NICCD early in infancy [14]. In this study, 36.7 % of patients had a chubby face, 90.0 % had AST/ALT >2 and 98.3 % had DB/TB<0.67, which is consistent with the previously reported diagnostic indicators and further confirms the value of these clinical and biochemical features in the early identification of NICCD. Consistently, 96.3 % of the patients in the present study exhibited elevated alkaline phosphatase (ALP), and 85.2 % of the patients presented with an ALP/ALT ratio > 10. Previous studies have demonstrated that elevated ALP and an ALP/ALT ratio > 10 can help distinguish NICCD from non-NICCD patients [15,16]. These findings further support the role of ALP and the ALP/ALT ratio as reliable auxiliary diagnostic markers for NICCD.

NICCD is characterized by abnormal plasma amino acid profiles, particularly a significant increase in citrulline, making it a primary biomarker for NBS. However, citrulline levels may not be elevated immediately after birth, resulting in the missed detection of several NICCD patients during NBS [17,18]. In the present study, only 5.2 % of patients had elevated citrulline on NBS, compared with 96.7 % at clinical presentation. The authors also observed increased methionine (83.6 %), arginine (78.8 %), tyrosine (11.8 %), and long-chain fatty acids (78.4 %), likely due to compensatory amino acid metabolism and impaired hepatic long-chain acylcarnitine metabolism secondary to hepatocyte energy insufficiency. These findings further confirm the metabolic characteristics of NICCD and the limitations of NBS, supporting the use of multi-indicator detection or adjustment of cut-off values for early diagnosis.

Citrin deficiency represents a genetic metabolic disorder with a global distribution, affecting individuals of various ethnic backgrounds. It has been reported in Asia, North America, and Europe, with a higher prevalence among patients in East Asia. To date, at least 140 pathogenic or likely pathogenic *SLC25A13* variants have been described [6]. c.852_855delTATG is the predominant variant in Thailand and Vietnam NICCD patients [6,16]. c.852_855delTATG, IVS16ins3kb, and c.1638_1660dup are the most common *SLC25A13* gene mutation loci in the Indian population [19]. The main high-frequency variations in Japan are c.1177+1G>A, c.852_855delTATG, c.1311+1G>A, which account for 60 % of the total mutations [20]. The most common variations in Korea are IVS16ins3kb, c.852_855delTATG and c.1177+1G>A [21,22]. In UK patients, c.1763G>A is the major pathogenic SLC25A13 gene mutation [23]. Song et al. identified c.852_855delTATG, IVS16ins3kb, c.1638_1660dup, and c.615+5G>A as the four most prevalent variants in China [12,13]. However, the number of patients in Zhejiang Province is limited. In the present study, the authors analyzed patients originally from Zhejiang province and found that c.852_855delTATG, IVS16ins3kb, and c.615+5G>A were the three most common mutation types. NICCD is inherited in an autosomal recessive manner, and it is generally caused by biallelic mutations in the *SLC25A13* gene. However, only a single heterozygous mutation was detected in four children in this study. Supported by the patients’ distinct clinical manifestations, specific alterations in blood metabolites and treatment responses, the diagnosis of NICCD was definitively verified. This may be attributed to factors such as the presence of large-fragment insertions or deletions in the homologous allele, as well as copy number variations, which cannot be detected by routine Sanger/NGS sequencing and panel sequencing.

The primary management for NICCD involves dietary intervention. Discontinuing breastfeeding and standard infant formula, and replacing them with a lactose-free, MCT-enriched formula, can lead to significant symptomatic improvement [24]. A large-scale Japanese follow-up study of 222 patients demonstrated that early dietary intervention improves both clinical symptoms and long-term prognosis [20]. A study by Abuduxikuer et al. of 61 patients diagnosed with NICCD found that poor prognosis was linked to delayed diagnosis and delayed treatment with lactose-free and/or medium-chain triglyceride-enriched (LF/MCT) formula [25]. In the present study, all patients were given dietary therapy after diagnosis or suspected diagnosis. After complementary foods were introduced, dietitians developed a specific protein-fat-carbohydrate ratio recipe for each patient, with 15–25 % protein, 40–50 % fat and 30–40 % carbohydrate recommended [24].

Of the 50 patients followed up, 94 % of patients achieved normal bilirubin levels within 6 months after discharge. Before dietary treatment, 26 patients (52 %) had growth retardation (HAZ and/or WAZ/BAZ < −2), while only 5 patients (10 %) remained growth-retarded at the most recent follow-up. These findings indicate the efficacy of dietary treatment. In addition, 68 % of patients showed a preference for foods rich in protein and lipid and/or an aversion to carbohydrate-rich foods. This special dietary habit may potentially represent a compensatory mechanism for the associated metabolic dysfunction [26].

Eleven patients still experienced episodes of hypoglycemia. Four patients had no hypoglycemic episodes after one year of age following the addition of MCT oil or intensified feeding, confirming the therapeutic effect of MCT in providing energy and improving hepatic metabolism [10]. Four patients experienced one hypoglycemic episode during morning fasting or illness, while one patient had two episodes. Two patients exhibited recurrent episodes of early-morning fasting hypoglycemia. Both failed to adhere to the dietary treatment and attend regular follow-up; one refused MCT oil supplementation, while the other used MCT oil irregularly. These factors may represent the key triggers for hypoglycemia recurrence in NICCD. After timely identification and treatment, no serious complications occurred. This highlights the importance of accurately identifying the clinical manifestations of NICCD and underscores the essential role of disease-related education for patients and their families.

The dyslipidemia associated with citrin deficiency is primarily attributed to two complementary mechanisms: the downregulation of the peroxisome proliferator-activated receptor alpha (PPARα) signaling pathway, which impairs fatty acid oxidation, and the activation of the glycerol-3-phosphate-carbohydrate responsive element-binding protein (G3P-ChREBP) pathway, which promotes hepatic de novo lipogenesis [27,28]. In this study, eleven patients presented with dyslipidemia, including nine with elevated total cholesterol, one with elevated triglycerides, and one with concurrent elevations in both total cholesterol and triglycerides. This may be related to poor dietary compliance, as excessive carbohydrate intake can exacerbate metabolic disturbances in citrin deficiency.

In the present study, three patients progressed to FTTDCD. All patients remained in stable condition without severe complications during the follow-up period. Most patients with NICCD have a generally good prognosis, but a small number of cases may develop severe complications, including liver failure, cirrhosis, hepatocellular carcinoma, necessitating liver transplantation, or even death [20,25,29]. Though symptoms resolve in some patients by adulthood, there remains a risk of progression to fatal CTLN2 decades later 20, 27. Therefore, long-term follow-up is essential for managing the prognosis of patients with NICCD, as it facilitates the early detection of abnormal changes and prevents disease progression.

The major limitation of this study is its retrospective design. Reliance on historical medical records and follow-up data inherently introduces the risk of information bias. Specifically, certain clinical data (including detailed dietary records and long-term treatment compliance) may have been incomplete or inaccurately documented, which could potentially confound the analysis of factors associated with disease progression and treatment outcomes. Furthermore, the patients were predominantly from Zhejiang Province, which may not represent the overall disease spectrum of citrin deficiency in China. Additionally, supplementary assays were not performed to detect potential secondary pathogenic variants in the homologous allele among these four patients.

In conclusion, the present study presents a retrospective analysis of the clinical, biochemical, genetic and follow-up data of patients with NICCD in China. Patients with NICCD exhibit a broad spectrum of clinical manifestations. c.852_855delTATG, IVS16ins3kb and c.615+5G>A are the mutation hotspots of the SLC25A13 gene in NICCD patients in Zhejiang, China. Early intervention with a specialized diet is crucial for effective management and a favorable prognosis. Education of pediatric patients and their families is of great importance.

## Funding

None.

## Data availability statement

The datasets are not publicly available due to ethical restrictions and institutional privacy policies. The data contains sensitive information that could compromise the anonymity of the participants. Requests to access the data should be directed to the corresponding author.

## CRediT authorship contribution statement

**Lingli Chen:** Conceptualization, Data curation, Formal analysis, Writing – review & editing. **Jingan Lou:** Project administration, Writing – review & editing. **Youyou Luo:** Project administration, Writing – review & editing. **Youhong Fang:** Resources, Writing – review & editing. **Mingfang Sun:** Data curation, Writing – review & editing. **Jindan Yu:** Conceptualization, Resources, Formal analysis, Writing – review & editing.

## Conflicts of interest

The authors declare no conflicts of interest.
